# A Case Report of Primary Melanoma of the Urethra

**DOI:** 10.7759/cureus.52018

**Published:** 2024-01-10

**Authors:** Heong Jin C Ahn, Arsh N Patel, Albert Tine, Srivikram Margam S, Parth K Patel, Todd J Kendall

**Affiliations:** 1 Department of Research, Alabama College of Osteopathic Medicine, Dothan, USA; 2 Department of Research, George Washington University School of Medicine and Health Sciences, Washington, D.C., USA; 3 Department of Pathology, Ascension Providence Hospital, Mobile, USA

**Keywords:** male urethra, urological malignancy, benign and malignant tumors, malignant melanoma of male urethra, melanoma

## Abstract

We illustrate a notable case of an 83-year-old male who presents to a community hospital with abdominal pain and hematuria. A few days after admission, an ulcerated lesion was found to be visible toward the ventral aspect of the penis, as well as bright red blood at the urethral meatus. An excisional biopsy of the urethral meatus, mid-urethra, and urethral tissue was done, and immunohistochemistry helped support the diagnosis of primary melanoma of the urethra. The pathophysiology and guidelines for treatment are discussed. Our purpose in putting forward this case is to present a rare diagnosis of primary melanoma of the male urethra and to emphasize the importance of early recognition to reduce the occurrence of invasive malignancy.

## Introduction

Primary malignant melanoma of the urethra, a type of mucosal melanoma, is an exceedingly rare neoplasm of melanocytes associated with high malignant potential. Mucosal melanomas themselves only comprise 1% of melanomas. Melanomas of the urinary tract only comprise 4% of mucosal melanomas [1]. The most common regions impacted in the genitourinary tract by malignant melanoma are the urethral meatus and distal urethra, although less commonly the bladder and ureter can be affected as well [2-4]. Unlike cutaneous melanoma, there is a lower incidence of BRAF mutations in mucosal melanomas; PI3K/Ak strain transforming (Akt)/mammalian target of rapamycin (mTOR) pathway alterations and c-kit mutations are more commonly observed in this group of neoplasms [5]. Poor outcomes for patients are typically due to the invasive nature and late diagnosis of the condition; the estimated three-year survival rate upon diagnosis is under 5% [1]. Since the incidence of the malignancy is very rare, this type of melanoma has not been thoroughly studied. The general diagnostic study of choice is biopsy, followed by immunohistochemistry using S-100, Human Melanoma Black (HMB-45), and melanoma antigen (MART-1) to aid in diagnosis [4]. Due to the current lack of literature, this case report aims to provide a unique case presentation for possible addition to a systematic review of this topic.

## Case presentation

We present an African-American 83-year-old male admitted to the hospital with complaints of abdominal pain and gross hematuria. He had a history of gross benign prostatic hyperplasia, hypertension, atrial fibrillation, and chronic kidney disease. His home medications included cephalexin, docusate sodium, doxycycline, ferrous sulfate, phenazopyridine, and tramadol. He was a former smoker with a one-pack-year history but quit many years ago and was an occasional user of alcohol.

He had a complicated urologic history of intermittent retention six months ago and was hospitalized previously for gross hematuria after the placement of a Foley catheter. He was asked to follow up with a cystoscopy as an outpatient procedure several weeks later. Approximately two weeks prior, the patient underwent a cystoscopy with clot evacuation and the placement of a suprapubic catheter.

At the time of presentation of gross hematuria, bloody discharge was noted from the penis. The suprapubic catheter was irrigated with the return of yellow urine. The urethral catheter was subsequently removed, and it was originally discussed with the patient that post-procedure oozing from the penis was expected.

On current hospital admission, the patient was found to have microcytic anemia, hyperkalemia, and acute kidney injury (Table 1). Urinalysis was positive for leukocyte esterase, suggesting an underlying urinary tract infection, and was treated with empiric antibiotics (Table 2).

**Table 1:** Complete blood count and complete metabolic panel

Lab	Patient value	Normal limit
Hemoglobin	8.3 g/dL	14-18 grams/deciliter (g/dL)
Hematocrit	25.9%	41-50%
White blood cells	10x10^9/liter (L)	4.5-11 x10^9/L
Neutrophils	78.3	40-60%
Mean corpuscular volume	74	80-100 fL
Mean corpuscular hemoglobin	23.7	Picograms (pg) per cell
Mean corpuscular hemoglobin concentration	32	32-36 g/dL
Platelets	262,000	150,000-450,000 microliter (uL)
Sodium	135	135-145 milliequivalents per liter (mEq/L)
Potassium	4.7	3.5-5.2 mEq/L
Chloride	105	96-106 mEq/L
Bicarbonate	24	23-28 mEq/L
Glucose	126	70-100 milligrams/deciliter (mg/dL)
Blood urea nitrogen (BUN)	27	7-20 mg/dL
Creatinine (Cr)	2.4	0.7-1.3 mg/dL
Estimated glomerular filtration rate (eGFR)	31	>60 milliliter/minute/1.73meter^2
Total protein	7	6.0-8.3 g/dL
Albumin	3	3.4-5.4 g/dL
Alkaline phosphatase (ALP)	99	44-147 international units per Liter (IU/L)
Alanine transaminase (ALT)	5	7-55 IU/L
Aspartate transaminase (AST)	12	8-33 IU/L
Anion gap	11	4-12 mEq/L
Prothrombin time (PT)	11.1	10-13 seconds (s)
International normalized raio (INR)	1.0	2.0-3.0
Partial thromboplastin time (PTT)	27.7	25-35 s

**Table 2:** Urinalysis

Test	Patient value	Normal limit
Appearance	Cloudy	Straw
Bacteria	Trace	None seen
Bilirubin	Negative	Negative
Blood	3+	Negative
Color	Brown	Clear
Glucose	Negative	Negative
Ketones	Negative	Negative
Leukocyte esterase	1+	Negative
Nitrite	Negative	Negative
pH	6.5	5-9
Protein	2+	Negative
Red blood cell	3141/high-power field (hpf)	0-3/hpf
Specific gravity	1.01	1.003-1.030
Urobilinogen	Normal	Normal
White blood cell	116/hpf	0-2/hpf

**Figure 1:**
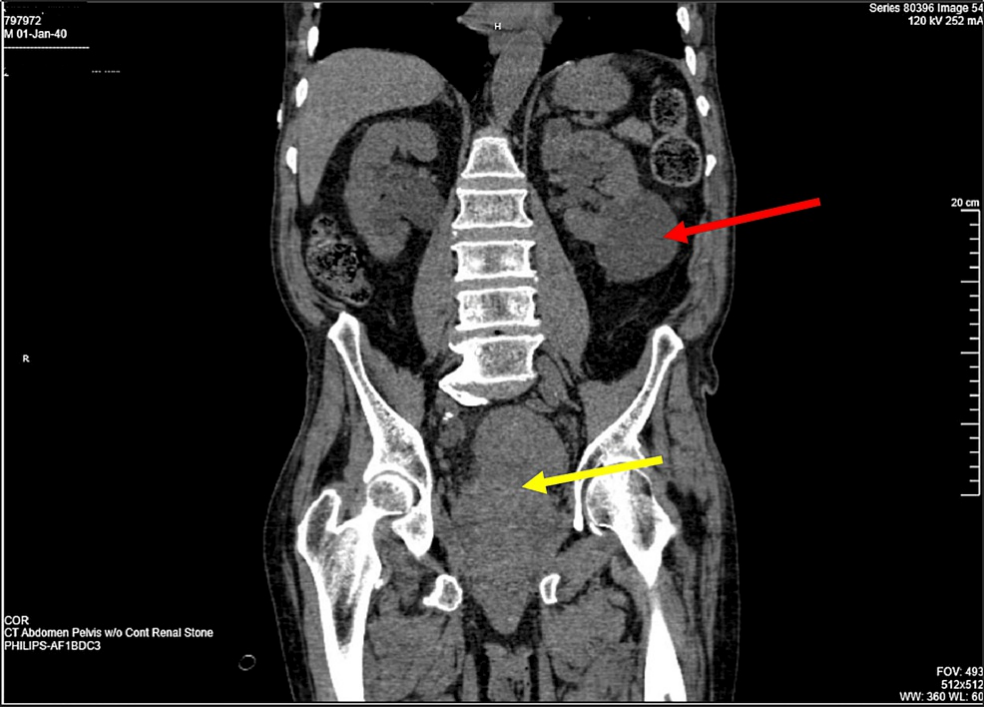
CT scan of abdomen and pelvis (coronal view) Moderate bilateral hydroureteronephrosis. Incidental finding of the left renal cyst within the lower pole (red arrow). Marked heterogeneity within the urinary bladder likely represents hemorrhage (yellow arrow)

On hospital day 6, urology was reconsulted as the patient experienced gross hematuria from the suprapubic catheter. A physical exam revealed bright red blood at the urinary meatus with a 2.0-centimeter (cm) ulcerated lesion visible toward the ventral aspect of the penis. A review of systems and other physical exam findings remained stable and unchanged throughout the course of hospital admission. A non-contrast computed tomography (CT) of the abdomen and pelvis was ordered to rule out renal calculi (Figure 1).

**Figure 2:**
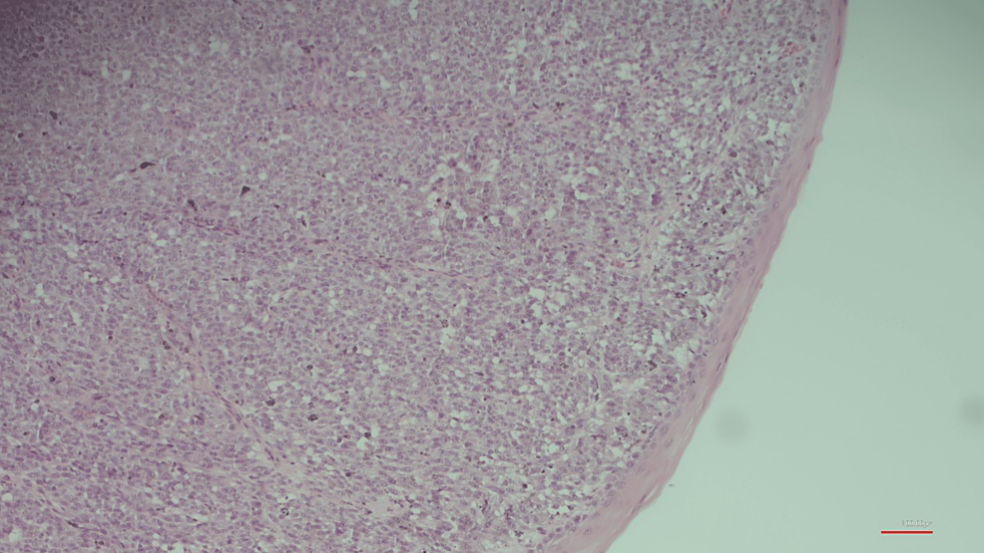
Poorly circumscribed lesion with marked cytologic atypia and epithelioid cells with prominent large, vesicular nuclei. Finely granular pigment noted throughout (H&E X10)

The CT showed moderate bilateral hydroureteronephrosis and heterogeneity throughout the urinary bladder, representing hemorrhage and air within the bladder likely due to catheter manipulation. An incidental finding of a left renal cyst was noted within the lower pole, measuring 7 cm. An excisional biopsy was ordered and performed at three locations: urethral meatus (4.7 cm), mid-urethra (4.1 cm), and urethral tissue (4.3 cm) (Figure 2). The gross pathological description of the biopsy was noted to have multiple areas of ulceration as well as dark brown pigment interspersed within the biopsy.

H&E staining showed a large area of epitheloid-like cells in the dermal layer with marked cellular atypia. Brown melanin pigment was scattered throughout the dermal spaces.

**Figure 3:**
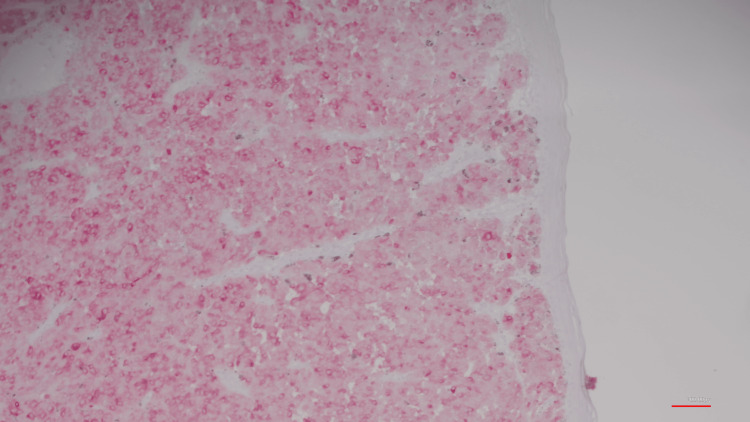
MART-1 stained cells (H&E X10)

All three biopsies returned immunoreactive with antibodies to MART-1 only and negative to vimentin and S100 (Figure 3). Unfortunately, the patient was lost to follow-up without any treatment or appropriate disposition.

## Discussion

The patient presented in the case report experienced gross hematuria, followed by a cystoscopic biopsy to obtain the diagnosis of primary malignant melanoma of the urethra. In general, mucosal melanomas are considered to be a more aggressive entity than cutaneous melanomas. Where cutaneous melanomas are known to exhibit a vertical growth pattern with a tendency to grow throughout the distinct levels of the epidermis, mucosal melanomas These cases reported in the literature have shown relatively poor prognosis due to delayed diagnosis or late presentation of symptoms. There has been only one systematic review to date completed by Khayyat et al., identifying 65 total patients from 47 manuscripts. The study showed molecular studies with varying alterations of c-kit, neuroblastoma ras viral oncogene homolog (NRAS), v-raf murine sarcoma viral oncogene homolog B1 (BRAF) (non-V600E and V600E), tumor protein p53 (TP53), and neurofibromatosis 1 (NF1) genes [5]. Few reports suggest using molecular or genetic studies for the differentiation of primary versus metastatic disease. From the review, outcomes were only shown in 52 of the cases, with 21 deaths, 10 patients without recurrence, two alive with disease, and five lost to follow-up [5]. Risk factors that have shown patients to be more susceptible to malignant melanoma include chemotherapy and immune-modulating therapies [5]. Interestingly, the patient described in this case report did not have any of these associated risk factors.

No set guidelines have been proposed for the diagnosis of primary malignant melanoma. Additionally, there has been no evidence suggesting urinary cytology as the cornerstone for the diagnosis of the neoplasm, but various studies have shown a positive correlation for a definitive diagnosis using urinary cytology and urethroscopy [6,7]. A CT scan of the whole body should be considered for staging purposes and to evaluate for distant metastasis.

The typical treatment for the vast majority of mucosal melanomas involves wide resection with lymph node dissection, followed by chemotherapy and/or immunotherapy [1]. However, there is no current consensus protocol for the treatment of urethral melanomas. Moreover, given the rarity of urethral melanomas and the lack of representation in the primary literature, developing innovative, targeted therapies has been a challenge [1]. A review of case reports suggests that urologists have two treatment options for improving patient outcomes: surgical intervention and chemotherapy. Select cases have suggested surgical management using partial urethrectomy, trans-urethral resection, local wide-excision of the urethra, radical cysto-urethrectomy with ileal conduit, or partial penectomy [4,6-13].

In terms of chemotherapy, dacarbazine-based regimens have been the historical mainstay treatment of genitourinary malignant melanoma and have been shown to have 5.6-7.8 months of median survival rates [5]. More recently, Yan et al. published the first phase two randomized control study using carboplatin plus paclitaxel and bevacizumab for advanced-stage untreated genitourinary malignant melanoma. The studies measured progression-free survival and concluded an increase from 3.0 to 4.8 months and overall survival from 9.0 to 13.6 months in the respective cohorts [5,14]. Advancement to a phase three study was recommended to confirm potential benefits.

## Conclusions

We illustrate an 83-year-old male who presented with abdominal pain and hematuria and was subsequently admitted based on these complaints. He had a six-month history of intermittent urinary retention and had been previously hospitalized for gross hematuria after the placement of a Foley catheter. During his hospital stay, a CT scan of the abdomen and pelvis was done, which showed moderate bilateral hydroureteronephrosis. A physical examination demonstrated a ventral aspect lesion on his penis, which warranted an excisional biopsy and resulted in the diagnosis of primary malignant melanoma of the urethra. The limited published reports in the literature may be due to underreporting, but this case report aims to add to the current literature. Additionally, we include the traditional treatment of genitourinary malignant melanoma and the importance of evolving targeted therapies based on molecular studies.
